# Reconstruction of composite regulator-target splicing networks from high-throughput transcriptome data

**DOI:** 10.1186/1471-2164-16-S10-S7

**Published:** 2015-10-02

**Authors:** Panagiotis Papasaikas, Arvind Rao, Peter Huggins, Juan Valcarcel, A Javier Lopez

**Affiliations:** 1 Department of Biological Sciences, Carnegie Mellon University, 5000 Forbes Avenue, 15213 Pittsburgh, PA, USA; 2 Current address: Centre for Genomic Regulation (CRG, c/Dr. Aiguader 88, 08003 Barcelona, Spain; 3 Universitat Pompeu Fabra, Dr. Aiguader 88, 08003 Barcelona, Spain; 4 Department of Computational Biology, Carnegie Mellon University, 5000 Forbes Avenue, 15213 Pittsburgh, PA, USA; 5 Current address: Department of Bioinformatics and Computational Biology, The University of Texas MD Anderson Cancer Center, 77030 Houston, TX, USA; 6 Current address: Robotics Institute, Carnegie Mellon University, 5000 Forbes Avenue, 15213 Pittsburgh, PA, USA; 7 Institució Catalana de Recerca i Estudis Avan¸cats (ICREA), c/Dr. Aiguader 88, 08003 Barcelona, Spain

**Keywords:** Splicing, Regulatory Network, Module, Exon, Graphical Model, mRNA Processing, Splicing Factor, Regulator, Development

## Abstract

We present a computational framework tailored for the modeling of the complex, dynamic relationships that are encountered in splicing regulation. The starting point is whole-genome transcriptomic data from high-throughput array or sequencing methods that are used to quantify gene expression and alternative splicing across multiple contexts. This information is used as input for state of the art methods for Graphical Model Selection in order to recover the structure of a composite network that simultaneously models exon co-regulation and their cognate regulators. Community structure detection and social network analysis methods are used to identify distinct modules and key actors within the network. As a proof of concept for our framework we studied the splicing regulatory network for Drosophila development using the publicly available modENCODE data. The final model offers a comprehensive view of the splicing circuitry that underlies fly development. Identified modules are associated with major developmental hallmarks including maternally loaded RNAs, onset of zygotic gene expression, transitions between life stages and sex differentiation. Within-module key actors include well-known developmental-specific splicing regulators from the literature while additional factors previously unassociated with developmental-specific splicing are also highlighted. Finally we analyze an extensive battery of Splicing Factor knock-down transcriptome data and demonstrate that our approach captures true regulatory relationships.

## Background

Alternative splicing is a widespread regulatory mechanism whose correct function is important for normal development and cellular behavior, and whose dysfunction plays an increasingly prominent role in the causation and modulation of human disease [1]. Basic splicing processes are fairly well understood, and progress has been made in defining several of the mechanisms and factors involved in alternative splicing regulation [2].

A large part of our current view of alternative splicing regulation is based on studies of single splicing events. These efforts have substantially advanced our understanding of the process, but they are often limited by their narrow scope and generalizing the conclusions is not straightforward. At the other end of the spectrum, high-throughput approaches (genome-wide knock downs, CLIP-related techniques) can provide more general information on splicing regulation; however, practical considerations typically constrain their focus to small groups of factors. Furthermore, computational approaches to solve "splicing codes" (e.g [3,4]) are mainly focused on genome-wide prediction of cis-regulatory elements. Validation of results and inferences from these approaches still requires slow and expensive low-throughput approaches.

Another limitation of current approaches is their static focus (e.g. the patterns observed in the presence or absence of a given factor in a given cell line or developmental stage). The dynamic nature of splicing regulation in different contexts remains a challenge, but is also a rich source of information for elucidating regulatory mechanisms. High-throughput analyses are now generating copious data on stage-and tissue-specific transcriptome composition but the problem remains of how to exploit and build upon such data to arrive efficiently at comprehensive regulatory models.

To address these challenges, we propose an approach based on the application of network reconstruction and analysis methods to high-throughput transcriptome data. Our approach derives a heterogeneous graphical model that simultaneously captures co-regulated groups of exons as well as their putative cognate regulators. This composite representation can be valuable for highlighting and assigning function to previously uncharacterized components of the system and for pinpointing novel regulatory relationships, while analysis of its topology can reveal organizational features such as central players that are critical for the flow of regulatory information or modules of densely interconnected units that correspond to meaningful functional groupings. As a proof of concept this strategy is applied to the Drosophila developmental data generated from the modENCODE project [5]. Detailed analysis of module and central component characteristics in the final model and the overlay with orthogonal annotation and functional data demonstrate the merits of our approach.

The splicing machinery, mechanisms, and known regulatory factors are well conserved between Drosophila and mammals, and the frequency and complexity of alternative splicing are comparable. Thus, the strategies developed in this study and the biological insights are transferable to more complex mammalian systems.

## Results and discussion

### Quantification of alternative splicing during Drosophila development from modENCODE tiling-array and RNAseq data

Alternative Splicing was quantified for 28 time points during fly development using the publicly available modENCODE transcriptome data [5,6]. The time points correspond to 12 embryonic stages taken at 2h intervals, 6 larval stages, 6 pupal stages, day 1 and 5 adult males, and day 1 and 5 adult females. First, individual exon Percent Spliced In (PSI, [7]) indices were calculated for all internal exons using the tiling array data for each developmental time point. Estimation is based on normalizing the average array signal over the exon, over robust estimates for total gene expression (see Methods for details). Out of the total 31,342 strictly internal exons, 2,855 exhibited appreciable PSI fluctuations during development (coefficient of variation cv=σ/μ>0.2).

Next, we obtained independent estimates of exonic PSIs by reanalyzing the RNAseq data for the same timepoints (~3.5 billion 76nt reads, ~125 million reads per condition on average). RNAseq-based PSIs were calculated as the ratio of inclusion-supporting junction reads over the sum of inclusion and skipping junction reads (see also Methods). We constrained our analysis to exons with sufficient junction coverage in the dataset (*≥*10 inclusion plus skipping junction reads for at least half of the developmental timepoints), resulting in estimates for <50% of the exons quantified using the first type of analysis. Of those, 1,277 appeared variable in development using the same criterion as before.

Comparison of the PSI values from the two types of analysis shows reasonably good correlation across developmental stages suggesting our methods yield reliable estimates of exon usage (Additional File 1: Figure S1). For the purposes of this study we decided to use the more populous dataset of fluctuating exons and the corresponding PSI-values coming from the tiling array based analysis. The PSIs of all development-variable exons were then centered and scaled. The resulting values reflect changes over the basal (average) developmental inclusion at each time point. Standardization of the events also ensures that only the shape and not the magnitude of the fluctuations is important during network reconstruction. The developmental profiles of both the raw and standardized PSIs are shown in Additional File 1: Figure S2.

In a next step we analyzed 421 RNA-Binding Protein (RBP) genes implicated in RNA processing according to Flybase annotation. Among these, we identified 349 genes with variable expression levels across development (*cv >*0.2). The standardized expression values of these genes were used along with the standardized PSIs to compute a single sample covariance matrix. In the case of highly correlated entries coming from the same transcription unit only one of them was selected, based on the L1-norm of its correlation vector, in order to minimize redundancies in the final network (see Methods). This dataset was used as input in order to reconstruct a composite network that captures both developmental splicing profiles and potential regulatory relationships between exon targets and RBPs.

### Reconstruction of a composite splicing regulatory network for fly development

The time series partial correlations of exon inclusion levels as well as RBP expression formed the basis for deriving a splicing regulatory network for Drosophila development. Our strategy for network reconstruction was based on the graphical lasso (glasso) algorithm [8] for sparse network estimation and is described in detail in Methods. Briefly, the composite sample covariance matrix described above was passed on as input to glasso for estimation of a sparse inverse covariance matrix, which directly corresponds to a unique undirected graph (see also Methods). The distinction between the two types of network nodes (fluctuating exons as targets and RBPs as putative regulators) was incorporated naturally as prior information in glasso by specifying a lower regularization parameter for regulator-to-exon edges and a higher one for exon-to-exon edges (see also Methods). This strategy encourages connections between potential regulators and alternative exons during network reconstruction and attempts to at least partially explain exon variability during development as a consequence of regulator variability.

**Figure 1:**
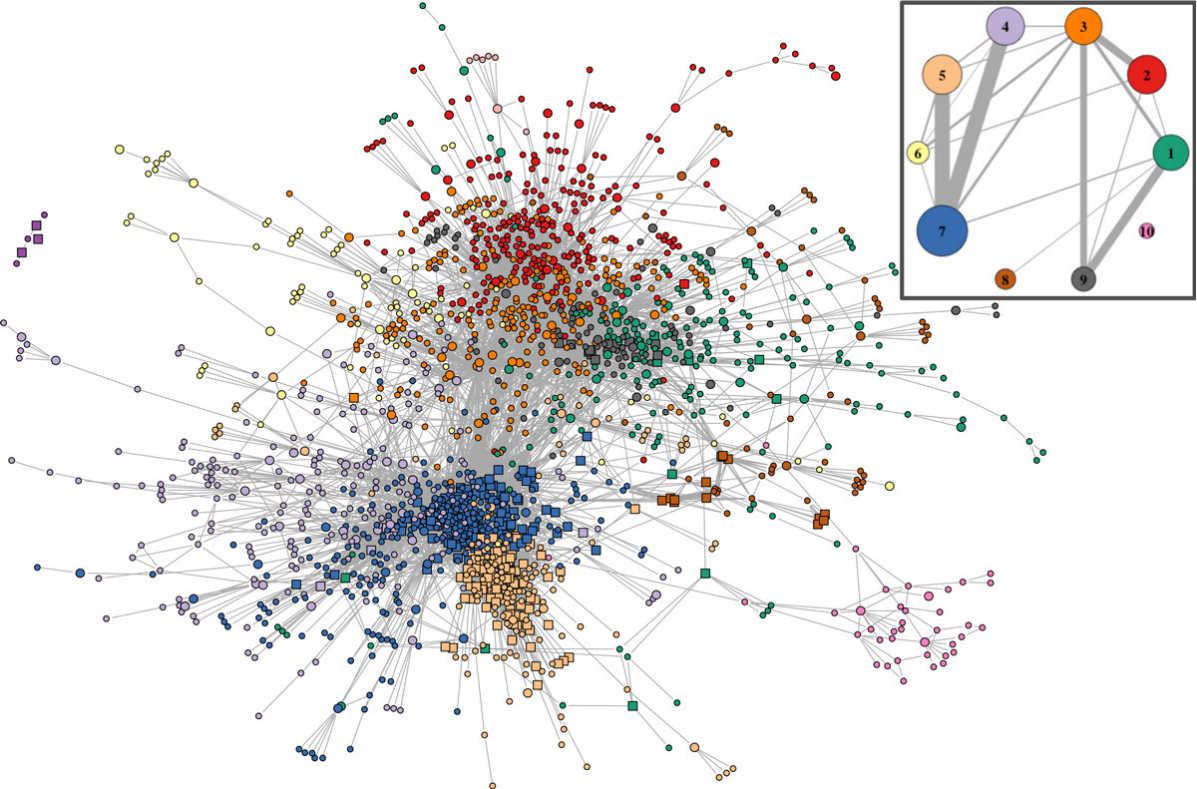
**Inferred Splicing Regulatory Network for Fly Development**. Only components that belong to modules with*>*5 members are shown. Circular and square nodes correspond to exon and RBP gene components respectively. Larger circles signify exons originating from RBP genes. Node coloring signifies membership to distinct modules. Inset: Inter-module connectivity in the inferred network. Circle area is proportional to module membership. Edge thickness is proportional to the fraction of observed exon-exon connections over the total possible connections among pairs of modules.

The resulting developmental splicing regulatory network is shown in Figure 1. The network contains 2,091 connected components of which 1,756 are exons and 335 are RBPs (see also Additional File 2 and Additional File 3). Gene Ontology analysis on the genes with exons partaking in the network reveals enrichment almost exclusively for terms related to both general and stage-specific organismal development and morphogenesis (Table 1). Interestingly, protein-binding is also among the top enriched GO terms suggesting that developmental progression involves extensive rewiring of protein-protein interactions via alternative splicing. These results indicate that our analysis successfully captures events involved in fly development and highlights the functional impact of alternative splicing in developmental progression.

**Table 1:** Identified exon modules display distinct functional profiles

Module	Top Enriched Go Terms(pval<1e-3)	Interacting Genes (pval<1e-3)	Top Regulators	Top Targets (non-RBPs)
1	instal larval or pupal morphogenesis, metamorphosis, post-embryonic morphogenesis	*DP, hp, Rbf, slpr, Myd88*	*Fib:2, CG6995:19, eIF-4a:6, sm:3, CG17838*	*CG17273:3, CSN3:4, CG9797:2, Abi:4, Akap200:5*

2	organ development, instar larval or pupal development, post-embryonic morphogenesis	*fz2, E2f, hop, hep, dco*	*Rbp2:3, Patr-1:3, dom:4, pum:3 CG7185:6*	*Trf2:4, CG3662:4, CG2943:7, Arf51F:4, CG2947:5*

3	regulation of cellular process, organ development, generation of neurons	*Su(H), Notch, mew, E2f, mys*	*(2)d:5 gw:52, CG30122:7 CG4119:3 Sxl:15*	*CG8116:2, CG42551:3, CG9132:2, Spt6:3, Gprk1:12*

4	organ development, locomotion, neuron di_erentiation	*Pc, mew, hop, ph-d, w*	*CG11266:4 su(w[a]):6 Dcp2:4, Zn72D:4, Psi:3*	*RhoGAP71E:5, CG4699:10, prod:2, Abl:6, l(1)G0232:6*

5	organ morphogenesis, sensory organ development, epithelium development	*rux, Pvf1, sli, Egfr, CycB*	*me31B:3, msi:6, CG11133, spn-E, xmas-2*	*CG12424:5, CG16791:13, tutl:6, LanB2:8, CG3907:3*

6	regulation of primary metabolic process, regulation of biosynthetic process, regulation of gene expression	*trx, w, Pc, arm, mor*	*CG6995:14 CG1646:10 Psi:5, brm:5, qkr58E-2:4*	*CG1244:10, CG42245:5, Syb:2 slmb:4, CG42614:12*

7	imaginal disc development, wing disc morphogenesis, post-embryonic appendage morphogenesis	*wupA, fz2, NiPp1, Tm2, Prm*	*CG34362:8, CG13124:10, shot:6, CG3209:4 CG8547:5*	*stv:8, CG15105:6, CG15105:9, CG34398:13, CG13188:6*

8		*Rac1, Rac2*	*Smg6:5, Chd1:2, snRNA:U5:38ABb, snRNA:U5:14B, snRNA:U5:23D*	*gammaCop:6, Nat1:2 Cip4:8, sec24:4, Utx:11*

9	sarcomere, myo_bril, tissue development	*Mhc, Dp, arm, l(2)gl, Rbf*	*Sxl:14, AGO1:6, Nop56:2, CG6209, SF2:3*	*CG42783:7, lqf:12, Zasp:10, CG42446:4, CG7188:2*

10		*Rho1, Pvf1, Taf1, Egfr, Ras85D*	*CG13124:7, CG4266:2, CG3558:4*	*CG15609:7, CG8677:4, CG2991:5, RhoGEF2:21, Pen:4*

Network	anatomical structure morphogenesis, system development, protein-binding, multicellular organismal development	*Sb, Rho1, Pc, mew, baz*	*CG30122:7, (2)d:5, pUf68:5, CG4119:3, gw:52*	*stv:8, psq:17, CG8116:2, l(1)G0232:6, trx:17*

The table shows the gene enrichment analysis, top interacting genes, putative regulators and targets for the 10 largest identified exon modules. GO analysis and analysis of genetic and physical interactions was carried out using the FlyMine database [33]. Top regulators and targets are listed in order according to their composite centrality scores.

### Identification of splicing regulatory modules using community structure detection

Network modules or communities can be defined loosely as sets of nodes with a more dense connection pattern among their members than between their members and the remainder of the network [9,10]. Module detection in real-world graphs is of considerable practical interest as they often capture meaningful functional groupings [9-11].

Here, we identify modules in the developmental splicing network using the greedy community detection algorithm [12] (see also Methods). The goal is to identify groups of exons that exhibit similar splicing regulatory patterns in the course of development and/or are under the control of the same regulatory circuitry. This analysis reveals a network community structure with a modularity value of 0.42, which indicates strong natural divisions in the network [13]. Both the identified network community structure as well as a simplified network topology illustrating the relationships between the largest modules are shown in Figure 1. RBP and exon components of these modules are listed in Additional File 4.

**Figure 2:**
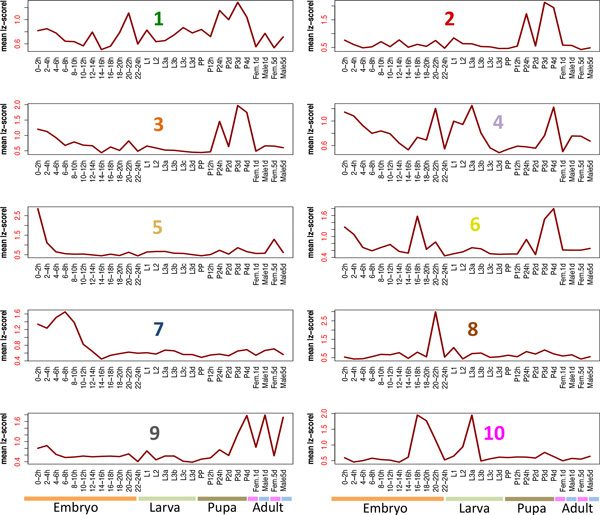
**The identified network modules exhibit distinct developmental profiles**. Y-axis shows the mean absolute scaled PSI for the exon components of each module. Module number coloring corresponds to the node colors in figure 1. Only profiles for the largest 10 modules are shown.

The identified exon modules exhibit distinct time series profiles of changes in exon inclusion (Figure 2) that are largely independent of the expression pattern of the originating genes (Additional File 1: Figure S3). The observed exon inclusion dynamics reveal mostly sharp inflections at specific time-points in development suggesting transient regulation. In order to obtain a comprehensive functional profile for each module we carried out a GO enrichment analysis within every module as well as an analysis of the top interacting module partners based on genetic and physical interactions (Table 1).

Modules 1,2 and 3 show similar peaks during pupal stages, although modules 1 and 3 present more complex splicing dynamics. All are enriched for terms related to instar larva or pupal morphogenesis and development. In addition modules 1 and 2 are both significantly enriched for interactions with genes required for dorsal closure (*hep, slpr*) as well as components of the DREAM complex (*E2f, Dp, Rbf*) which regulates transcription of developmentally controlled targets, whereas module 3 is specifically GO-enriched for neuron generation and for interactions with components of the Notch pathway (*Notch, Su-H*), which regulates neurogenesis in the central nervous system and the imaginal disks. Modules 4 and 6 present inflections during late pupal and mid-to-late embryonic stages and are both enriched for interactions with polycomb group (PcG) components (*Pc, ph-d*). Module 4 is nevertheless distinct in that it also shows a pronounced switch during the L2 to L3 larval transition and is highly enriched for terms related to locomotion and neuron differentiation. On the other hand, module 6 has additional interactions with genes (*trx, mor*) of the PcG-antagonisitic trithorax group (trxG) and is almost exclusively enriched for GO-terms related to metabolic processes.

The densely connected modules 5 and 7 show profiles characteristic of maternally loaded transcripts, with larger peaks at the beginning of embryonic development and less pronounced ones in the 5-day female. However, module 5 dynamics suggest rapid degradation prior to cellularization (2-3h post-fertilization) while module 7 isoforms persist past the maternal-to-zygotic transition (completed ~3h post-fertilization). In addition, module 7 is mostly enriched for GO-terms related to imaginal, wing-disc and appendage morphogenesis and for interactions with genes involved in muscle filament generation and contraction (*wupA, Tm-2, Prm*).

Modules 8 and 10 are exceptional in that they show no clear GO-term enrichment despite the fact that they exhibit simple splicing dynamics. These modules mostly contain peripheral, loosely connected nodes suggesting an ancillary role in development. Finally module 9 shows a distinctive male-specific profile with a switch immediately prior to metamorphosis. It is enriched for biological functions related to anatomical structure development and for components of the sarcomere and contractile fibers suggesting a role in the sexually-dimorphic fly musculature ([14], see next section).

### Inference of global and within-module splicing regulators using centrality measures

Centrality measures are structural attributes of network nodes that express the importance of their location within the network topography and/or how they influence network information flow [15]. These measures are typically used in the analysis of Social Networks but they have also been applied in studies of biological networks (see e.g [11,16-19]. Here we employed three different measures of centrality, namely Closeness [20], Betweenness [20] and PageRank [21] that capture different structural properties of the network nodes.

The closeness measure is based on geodesic distance between a node and all other nodes in the network. A node that is closeness-central can quickly interact with all the others, not only its neighbors [22,15]. Betweenness measures how often a node participates in the shortest paths that connect pairs of other nodes in the network. A node with high betweenness can control the flow of information, and may also serve as a liaison between distant regions of the network [22,15]. PageRank is a measure that quantifies the relative importance of individual nodes [21]. Nodes with high PageRank centrality have high visibility, meaning that they are often traversed, irrespectively of which path is chosen. As a result, perturbations in PageRank-central nodes can affect and propagate to large parts of the network.

**Figure 3:**
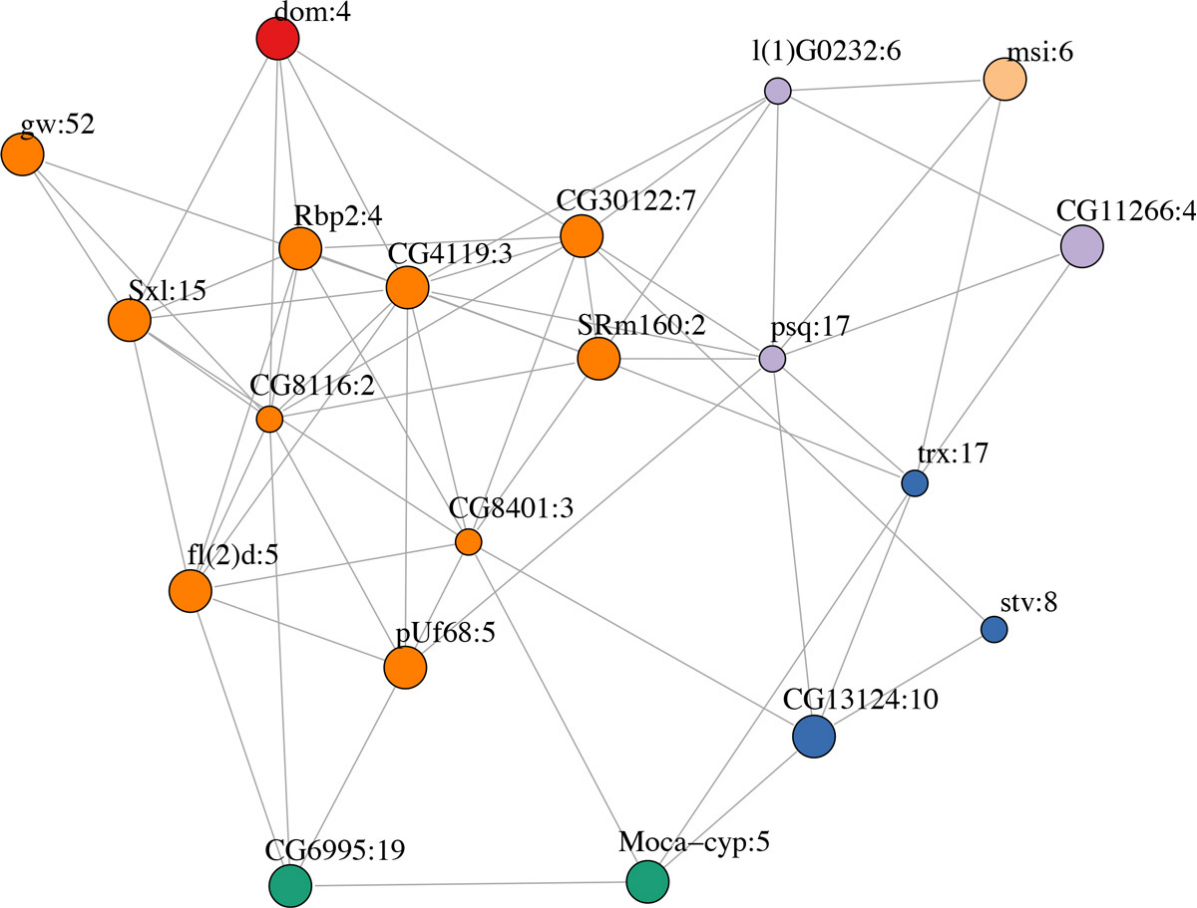
**Core sub-network containing the 20 central-most network nodes**. Different colors correspond to node members of distinct modules. Larger nodes depict putative regulators. Node coloring signifies the originating module (color assignment as in figure 1).

**Figure 4:**
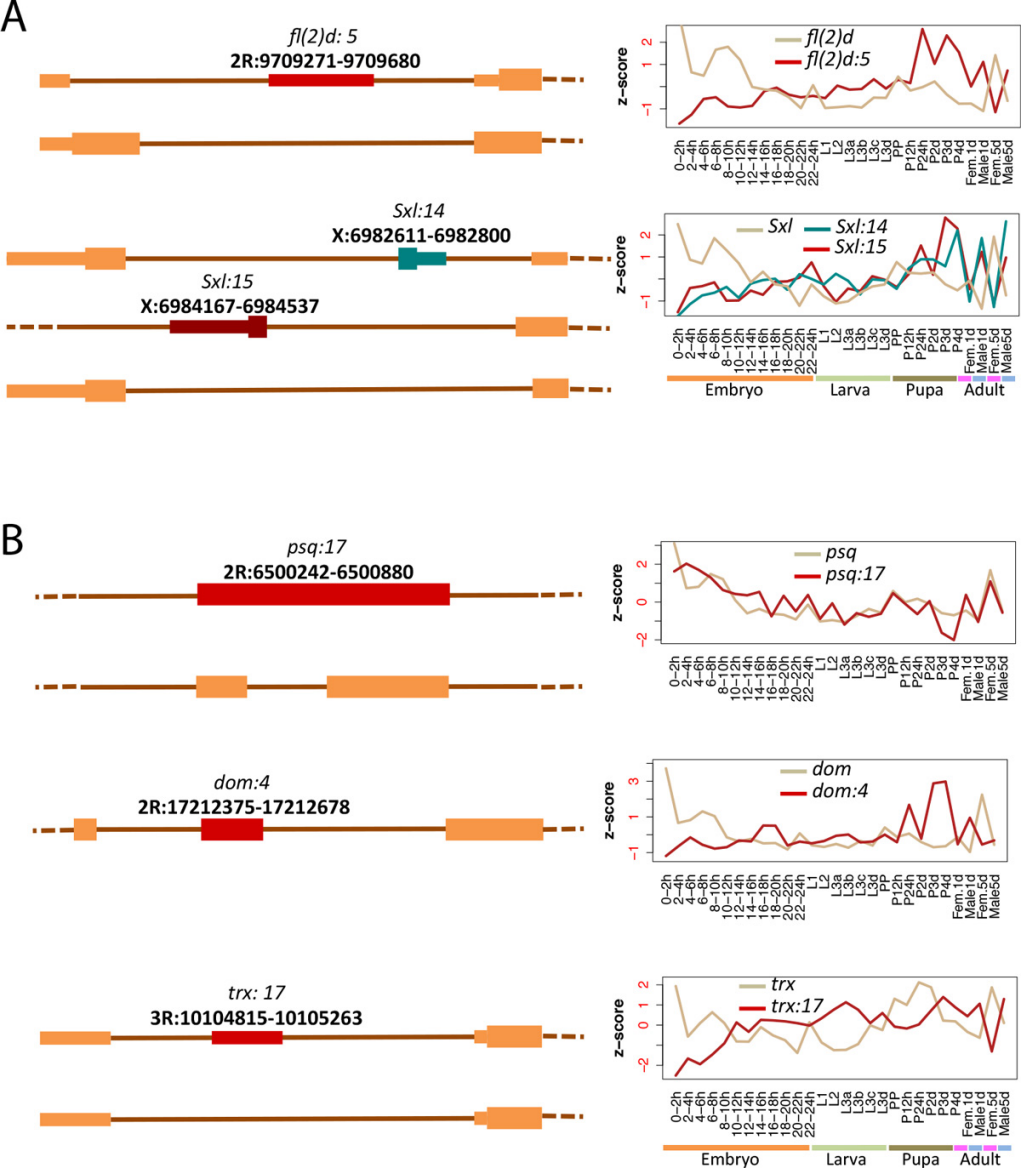
**Examples of key exons in fly development according to their centrality scores**. Transcript structure around several identified critical network exons (left) and their corresponding inclusion and gene expression time-series profiles (right). Exons known to be important for sex differentiation (A) and exons in genes involved in chromatin remodelling (B). Transcripts are shown in 5'-3' orientation irrespectively of strand. Lines denote introns, thick and thin boxes denote coding and non-coding (untranslated) exonic regions respectively.

We assigned as a measure of node importance the average rank of the node using the three aforementioned centrality criteria. We applied this centrality analysis first to the complete network in order to highlight core network components with effects that ramify throughout development (Figure 3, Table 1) and then to the individual network modules to identify locally acting regulators (Table 1). Among the global regulators several are exons of RBPs known to be involved in developmental regulation (*pUf68, gw, Sxl, fl(2)d, msi, SRm160*). Of particular interest are the exons of the known sex differentiation master regulators *Sxl *and *fl(2)d*. Two of these exons, *fl(2)d:5 *and *Sxl:15*, are part of the core network and are also among the top 5 candidate regulators of their respective module (module 3, Table 1). *fl(2)d:5 *is a non-coding 5'UTR exon whose skipping leads to the addition of 124aa in the N-terminus of the protein and the production of a functional isoform required for female-specific *Sxl *splicing (Figure 4A)[23]. This event is connected to *Sxl:15*, a cassette exon upstream of the well-characterized *Sxl *male-specific exon. Inclusion of *Sxl:15 *leads to a longer protein isoform based on annotation (Figure 4A). Both *fl(2)d:5 *and *Sxl:15 *appear to be male-specific, peaking during mid-to-late pupal stages. The characterized *Sxl *male-specific exon, *Sxl:14*, also appears as the top regulator of its module (module 9, Table 1) and has a male-specific profile. Several studies have shown that components of the *Sxl *cascade such as *dsx *and *fru *are involved in multiple aspects of behavioral and somatic sex-differentiation including motor-neuron-dependent muscle formation. The functional profiles of modules 3 and 9 linking them to neurogenesis and muscle development are consistent with roles in establishment of neuromuscular sexual dimorphism during late development.

Interestingly 3 of the 6 non-RBP components of the core network are exons of the PcG and trxG chromatin remodeling complexes (*trx:17, psq:17, dom:4 *) and for two of these cases (*trx:17 *and *psq:17 *) developmental specific alternative splicing is also supported by RNAseq data. In *psq:17 *retention of a short intron results in introduction of 18aa overlapping the first Helix-Turn-Helix domain of the protein (Figure 4B). In *trx:17 *exon inclusion results in a longer 5'UTR which is associated with lower expression values (Figure 4B) suggesting less efficient transcription and/or decreased mRNA stability. These results highlight the interplay between splicing and chromatin regulation as key for the establishment of differential gene expression profiles that underly fly development.

### Knock-downs of RBPs show significantly enhanced effects on their predicted targets

**Figure 5:**
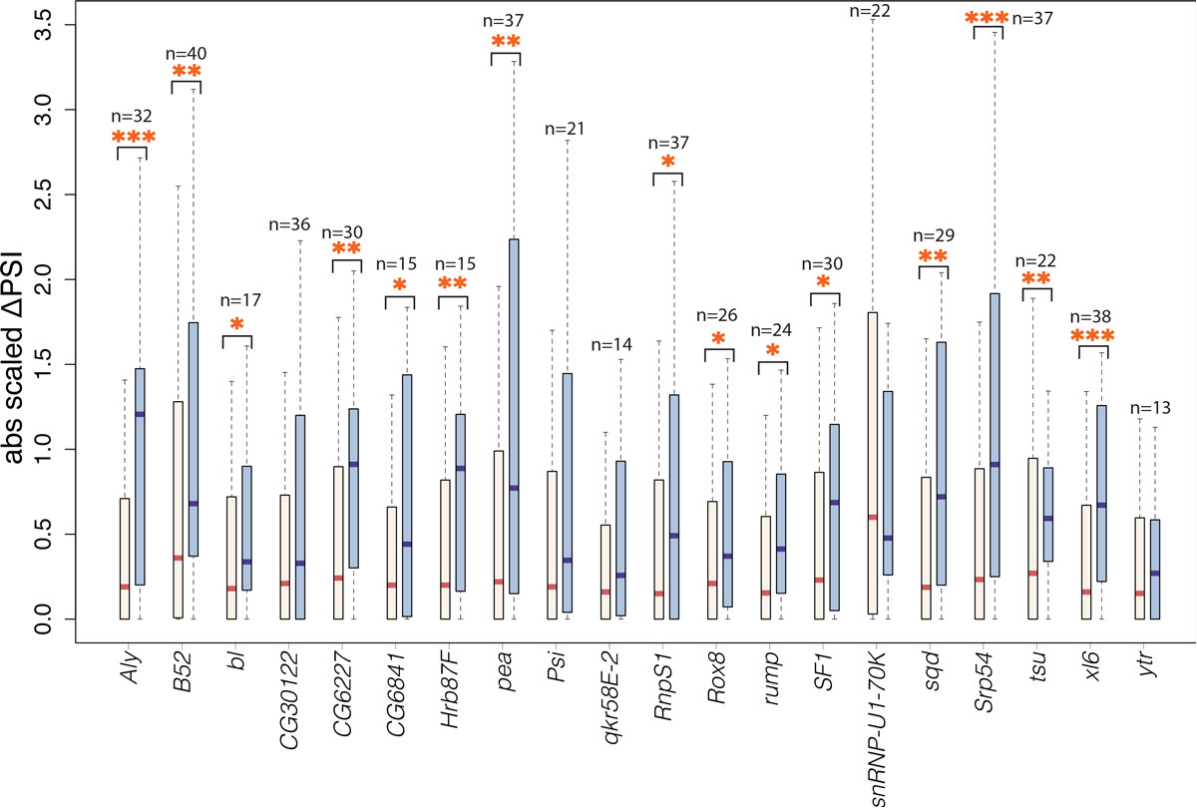
**RNAi KDs of RBPs in a heterologous context show increased effects on their predicted targets**. Boxplot summarizing the effects of RBP knock-downs in S2 cells on their target (blue) vs their non-target (beige) developmental network exons. Stars indicate significance of difference in the effects in the two sets of exons (Wilcoxon rank-sum test, * p-val<0.1, ** p-val<0.01, *** p-val<0.001 ). The number of targets *n *for each RBP is shown. The number of non-targets for each RBP is 1092-*n*. Only RBPs with *≥ *10 targets in the network are shown

In order to confirm that our network captures *bona-fide* regulator-target relationships we analyzed the dataset of fly splicing factor RNAi knockdowns available from modENCODE. RNAseq data are available from the consortium for knock-downs of 58 RBPs plus Untreated samples in drosophila S2 cells. In total 2.6 billion reads (~45 million reads per condition) were mapped and analyzed. From these data we derived PSI indices for all exons present in our developmental network for each RNAi knock-down (see Methods). Next we filtered out exons that belong to genes that are not expressed in S2 cells and/or are not affected by any of the 58 knock-downs suggesting that these exons are not differentially regulated within the S2-cell line context. Within the remaining set we compared the effects of each RBP knock-down on the developmental target vs non-target exons according to our inferred network. We consider as putative developmental targets of an RBP those exons that are directly connected to the RBP gene or to one of its fluctuating exons in the network. Conversely, non-targets are exons of the same final filtered exon set not directly connected to any network components of the RBP. The effect of each KD to every exon was summarized as the absolute scaled ΔPSI value between KD condition and untreated samples. Our analysis shows that the RBP targets inferred from the developmental network are consistently (19/20 RBPs) and in most cases significantly (Wilcoxon rank sum pval<0.1 for 15/20 RBPs, combined pval<1e-20) perturbed at higher levels compared to their non-target counterparts upon RBP knock down in the S2 cells (Figure 5). This result strongly suggests that our network captures true regulatory relationships, though we note that we cannot discriminate between direct and indirect effects.

## Conclusions

In this work we presented a method for the reconstruction of composite splicing regulatory networks that can recover simultaneously both modules of co-regulated exons as well as regulatory relationships between regulator (RBPs) and target (exons) components. Reconstruction is based on the estimation of a sparse covariance matrix that includes both types of components using a regularization approach that naturally incorporates putative regulatory relationships as prior information in the form of non-uniform regularization parameters. Our analysis on the characteristics of the derived exon modules suggests that exon groupings undergoing coordinated splicing regulation reflect coherent functional groupings implicated in distinct stages and processes during fly development. This observation indicates that the obtained exon modules can be used as proxies for inferring the function of non-characterized network components. In addition, experimental knock-down of splicing regulators produces larger effects on their computationally inferred targets as opposed to non-targets, indicating that this approach is also valuable as a hypothesis generator for regulatory relationships.

For this study only two possible values of the regularization parameter were used (see Methods) in order to specify two possible types of network relationships (regulator-target and everything else). However it is straightforward to modify this approach to incorporate more elaborate patterns of prior knowledge based for example on enrichment of RBP binding motifs near the exons or available binding or functional data for subsets of the regulators. In addition, the same approach can also be directly applied for deciphering and contrasting the configuration and organizational properties of the splicing regulatory circuitry involved in different organisms and biological processes including mammalian tissue-specific splicing or cell differentiation and reprogramming. Finally the computational framework described here can easily be extended to other layers of gene expression regulation (e.g alternative promoter selection of polyadenylation, mRNA export and translation) as it is only limited by the availability of suitable datasets.

## Methods

### Pre-processing of tiling array data and array-based PSI estimation

The publicly available modENCODE tiling array data consist of signal values for 25mer probes covering the entire fly genome. The tiling array probes have an average center base spacing of 38 bp and are designed to be identical to the plus strand of the genome sequence. Three biological replicates were hybridized for each experiment. The data from replicate arrays are quantile-normalized and all arrays are scaled to median array intensity. Prior to data analysis unreliable probes are removed based on their variance and mean values. In particular a probe p is removed if its coefficient of variation *cv_p_*= *σ_p_/µ_p_ >*5 or its mean value *µ_p_ < −*200 or *µ_p_ >*20000.

Gene signals *S*(*G*) are calculated as the average of all probes that cover the gene regions that are constitutively included in the mature RNA as these are defined based on Flybase and modENCODE gene models (MB8 and MB9). Exon signals *S*(*E_i_*) are calculated as the average of tiling array probes covering the genomic coordinates of exon *i*. Next, exon inclusion indices for each developmental time point are determined for every exon as the fraction *S*(*E_i_*)*/S*(*GE_i_*).

Missing values are assigned to exon inclusion indices in cases where a reliable estimate cannot be acquired due to low gene expression or sparse probe coverage. Exons with *>*50% of missing values are discarded from subsequent analysis. Missing values for the remaining exons are filled in using 10-nearest neighbors imputation ([24], R function impute available as part of the bio-conductor project at http://www.bioconductor.org/). Finally both exon inclusion and gene expression time series data were scaled and centered.

### Pre-processing of RNAseq data and RNAseq-based PSI estimation

FASTQ files for the RNAseq data for both developmental time-points and RNAi treatments were downloaded from the modENCODE ftp repository (ftp://data.modencode.org/). For every sample the reads were mapped using the STAR aligner v2.4.0 and a two-pass mapping procedure [25] with standard ENCODE mapping parameters as those are detailed in the software manual. Genome sequence files and annotation were based on the dm3 assembly freeze and the corresponding flybase annotation [26] available from the UCSC table browser. For PSI calculation only junction spanning reads were used and the index was calculated as described in [7]. Missing values were assigned to Exons with less than 10 total inclusion and skipping junction reads per sample. Finally, missing value imputation and data scaling and centering was carried out as described above.

### Selection of representative transcription-unit events

Often events that belong to the same transcription unit are highly correlated and can form dense clusters in the final network. Typically this type of connections is highly redundant as it is the product of probes that interrogate overlapping gene segments, alternative events that are regulated as units (e.g mutually exclusive exons, jointly skipped exons) and methodological or biological biases (e.g incomplete annotation, NMD), in the estimates of gene expression in the presence of particular events. In order to minimize such redundancies in our superset of events *V *prior to network reconstruction, only one event per highly correlated same-transcription unit cluster is retained. In particular let *S⊂V *be a set of same-transcription unit events, each with at least one within-set high pairwise correlation (cor(i,j)>0.6,i,j∈S). Then, we select as set representative the event *e∈S *with the largest *L*1-norm of its top *k *correlations outside *S*. That is:

$$e=\text{arg}\underset{s\in S}{\text{max}}\sum \overset{k}{\underset{v\in V-S}{\text{sup}}}|cor\left(s,v\right)|$$

For this work we set *k *= 10.

### Graphical model selection for network reconstruction using graphical lasso

Undirected graphical models (UGMs) such as Markov random fields (MRFs) represent real-world networks and attempt to capture important structural and functional aspects of the network in the graph structure. The graph structure encodes conditional independence assumptions between variables corresponding to nodes of the network. The problem of recovering the structure of the graph is known as model selection or covariance estimation. In particular, let *G*=(*V, E*) be an undirected graph on *p*=*|*V*| *nodes. Given *n *independent, identically distributed (i.i.d) samples of *X *= (*X*1,..., *Xp*), we wish to identify the underlying graph structure. Restricting the analysis to Gaussian MRFs, the model assumes that the observations are generated from a multivariate Gaussian distribution *N *(*µ*, Σ). Based on the observation that, in the Gaussian setting, zero components of the inverse covariance matrix Σ*−*1 correspond to conditional independencies given the other variables, different approaches have been proposed in order to estimate Σ*−*1. Graphical lasso (glasso) [8] provides an attractive solution to the problem of covariance estimation for undirected models, when graph sparsity is a goal. The glasso algorithm has the advantage of being consistent, i.e. in the presence of infinite samples its parameter estimates will be arbitrarily close to the true estimates with probability 1. L1 regularization (lasso) is a smooth form of subset selection for achieving sparsity. In the case of glasso the model is constructed by optimizing the log-likelihood function:

$$\text{log}\;\text{det}\;\Theta -tr\left(S\Theta \right)-r||\Theta |{|}_{1}$$

where Θ is an estimate for the inverse covariance matrix Σ*−*1, *S *is the empirical covariance matrix of the data, ||Θ|| 1 is the *L*1 norm of Σ*−*1, and *r *is a regularization parameter. The solution to the glasso optimization is convex and can be obtained using coordinate descent. As noted in the original graphical lasso paper [25], it is possible to modify the glasso objective function in order to accommodate different regularization parameters for each variable. In that case the objective function can be rewritten as:

$$\text{log}\;\text{det}\;\Theta -\text{tr}\left(S\Theta \right)-||\Theta *R|{|}_{\text{1}}$$

where *R *is a *p × p *regularization parameter matrix and *∗ *indicates component-wise multiplication. This formulation provides a natural way to incorporate in glasso prior information on the network structure [27]. In this application we take advantage of this formulation and define a bimodal regularization matrix *R *that biases for edges between alternative exons and potential splicing regulators. In particular, our network is a mixture of two distinct types of variables: Variables of type *A *correspond to alternative exons that show differential inclusion between at least two developmental stages. Variables of type *B *correspond to candidate regulating elements. These can be either genes or exons of genes that are known or predicted to be implicated in RNA processing according to Flybase annotation AND show differential expression (in the case of genes) or inclusion (in the case of exons) during development. Connections between type *A *and type *B *variables are assigned a regularization parameter *r*1 and all other type of connections are constrained by a regularization parameter *r*2 *> r*1. Optimal choice of the regularization parameter for covariance estimation is an open theoretical problem [28-30]. In general, since the regularization parameter controls for type I errors in the network, it can be relaxed as the variance of the variables decreases [29]. For this application we set *r*1 to 0.75 and *r*2 to 0.85. We note however, that the social network analysis results presented here (identified modules, centrality analysis) are robust to different choices of values for *r*1, and *r*2 as long as *r*2 *> r*1 (data not shown). The glasso process for network reconstruction was implemented using the glasso R package by Jerome Friedman, Trevor Hastie and Rob Tibshirani (http://statweb.stanford.edu/~tibs/glasso/).

### Modularity-based community detection and measures of centrality

For a particular network partition *p*, the network modularity *M *(*p*) is defined as:

$$M\left(p\right)=\overset{m}{\underset{k=1}{\sum }}\left[\frac{l_{k}}{L}-{\left(\frac{d_{k}}{2L}\right)}^{2}\right]$$

where *m *is the number of modules in *p, l_k_* is the number of connections within module *k, L *is the total number of network connections and *d_k_* is the sum of the degrees of the nodes in module *k*. Here, we identified modules of exons that exhibit similar profiles across development by maximizing the network's modularity using the greedy community detection algorithm [12] implemented in the fastgreedy.community function of the igraph package [31], http://igraph.org).

Extensive definitions and algorithmic details for the computation of Closeness and Betweeness centralities and the Pagerank index can be found in [32]. All functions for centrality measure calculation are available through the igraph library ([31], http://igraph.org).

## Competing interests

The authors declare that they have no competing interests.

## Authors' contributions

P.P, A.R, PH and A.J.L conceived the study P.P wrote code, analyzed the data and wrote the manuscript. A.R and P.H edited the manuscript J.V provided conceptual advice and edited the manuscript A.J.L supervised the study and edited the manuscript.

## Acknowledgements

We acknowledge support of the Spanish Ministry of Economy and Competitiveness, ‘Centro de Excelencia Severo Ochoa 2013-2017’, SEV-2012-0208. Work in JV laboratory is supported by Fundación Botín, by Banco de Santander through its Santander Universities Global Division, Consolider RNAREG, Ministerio de Economía y Competitividad and AGAUR.

## Supplementary Material

Additional File 1 This file contains Supplemental Figures S1 to S3 and their legends Click here for file

Additional File 2 **This file contains in tab separated format the complete inverse covariance matrix that specifies the structure of the recovered splicing network**. Non-zero off-diagonal entries correspond to edges in the graph. Click here for file

Additional File 3 **This file contains annotation information for all the network components: Lists of the exon and gene IDs and symbols of the network components as well as genomic coordinates of the exon components in BED format**. Click here for file

Additional File 4 **This file lists the gene and exon members for each of the 10 described network modules**. Click here for file

## Declarations

Publication charges for this work were jointly funded by Fundacion Botin, Banco de Santander through its Santander Universities Global Division, Consolider RNAREG, Ministerio de Economia y Competitividad and AGAUR and the corresponding author's institution.

This article has been published as part of *BMC Genomics *Volume 16 Supplement 10, 2015: Proceedings of the 13th Annual Research in Computational Molecular Biology (RECOMB) Satellite Workshop on Comparative Genomics: Genomics. The full contents of the supplement are available online at http://www.biomedcentral.com/bmcgenomics/supplements/16/S10.
