# Supplementary material for: Reconstruction of composite regulator-target splicing networks from high-throughput transcriptome data
Source: BMC Genomics. 2015 Oct 2;16(Suppl 10):S7. doi: 10.1186/1471-2164-16-S10-S7 (PMC4603746; doi:10.1186/1471-2164-16-S10-S7)
Supplement: Additional File 1 — This file contains Supplemental Figures S1 to S3 and their legends [file 1471-2164-16-S10-S7-S1.pdf]

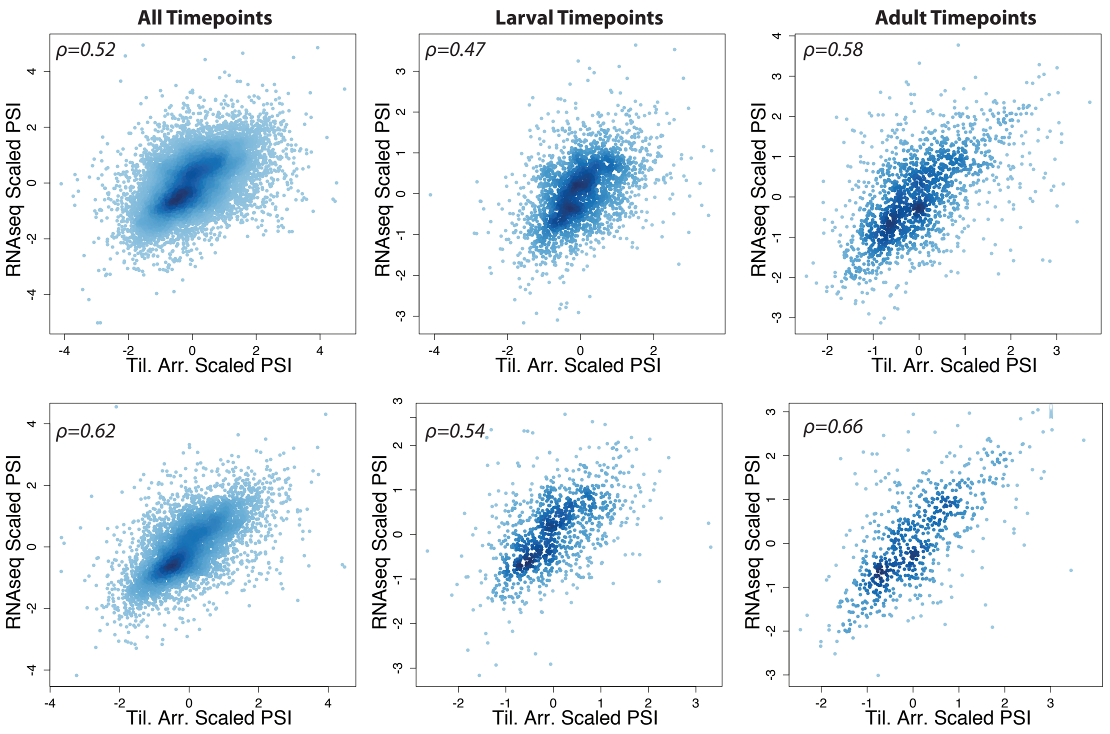

Figure S1: Correlation between Tiling array and RNA-seq based PSI estimates. Scatterplots are shown for all variable exons (top) and variable exons that are members of the final network (bottom). Larval and Adult timepoints show the lowest and highest correlation between the two quantifications respectively and are shown separately. ρ indicates Spearman's correlation.

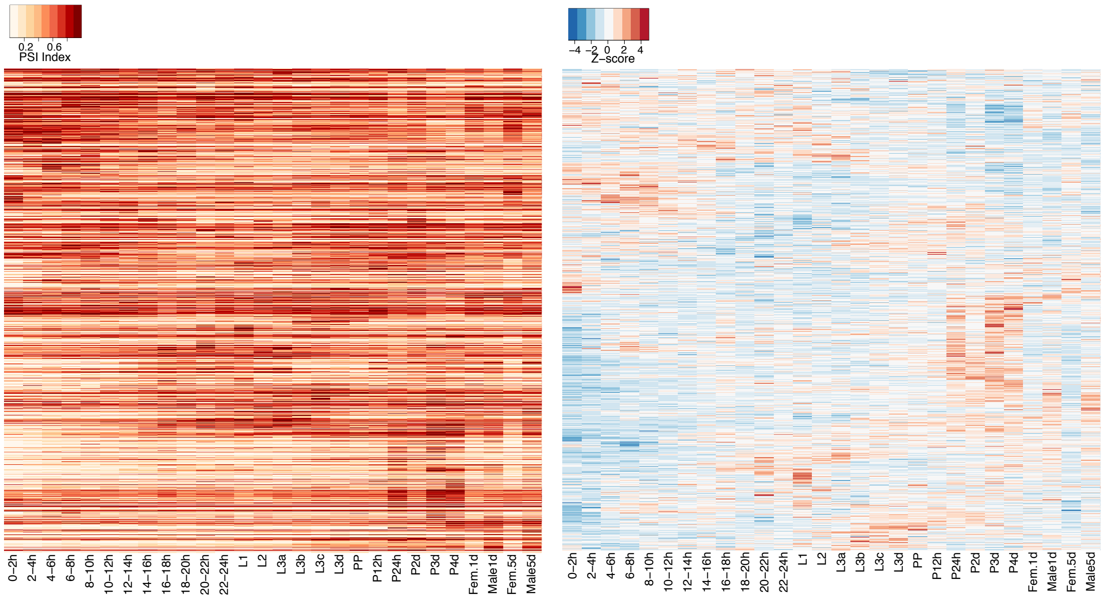

Figure S2: Heatmaps showing the unscaled (left) and scaled (right) PSI for all variable internal exons according to tiling-array quantification. Developmental stages are indicated at the bottom. Data are clustered across the Y-dimensions (ward linkage, similarity measure based on Pearson correlation).

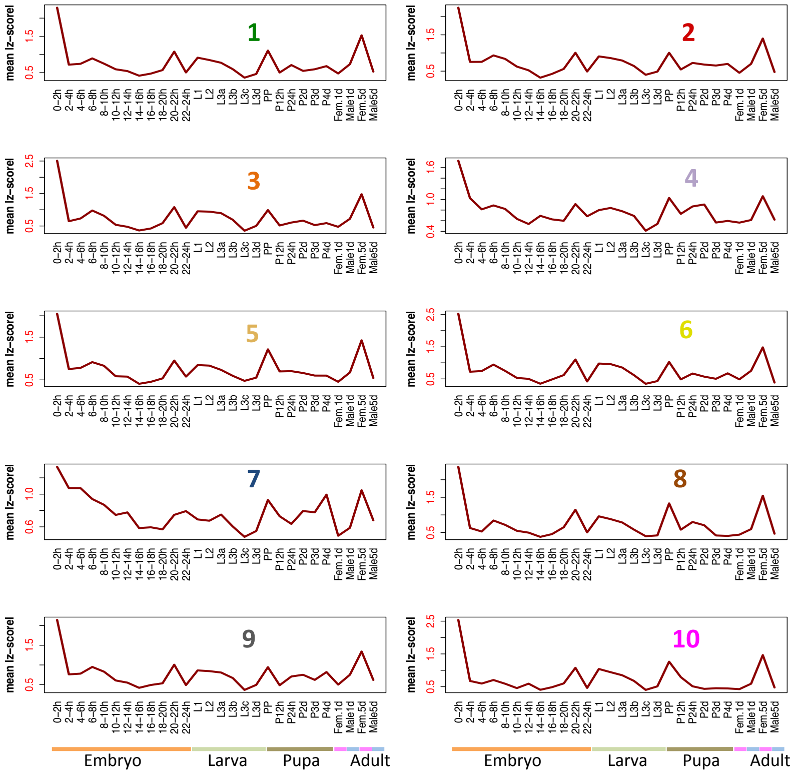

Figure S3: Time series profiles for the expression of the originating genes of the exon components of each network module. Y-axis shows the mean absolute scaled expression. Module number coloring corresponds to the node colors in figure 1. Only profiles for the largest 10 modules are shown.
